# Prognostic implications of forkhead box protein O1 (FOXO1) and paired box 3 (PAX3) in epithelial ovarian cancer

**DOI:** 10.1186/s12885-019-6406-6

**Published:** 2019-12-10

**Authors:** Gwan Hee Han, Doo Byung Chay, Sanghee Nam, Hanbyoul Cho, Joon-Yong Chung, Jae-Hoon Kim

**Affiliations:** 1 Department of Obstetrics and Gynecology, Yonsei University College of Medicine, Seoul, Republic of Korea; 2 Department of Obstetrics and Gynecology, Gangnam Severance Hospital, Yonsei University College of Medicine, 211 Eonju-Ro, Gangnam-Gu, Seoul, Seoul 06273 South Korea; 3 Experimental Pathology Laboratory, Laboratory of Pathology, Center for Cancer Research, National Cancer Institute, National Institutes of Health, Bethesda, MD USA

**Keywords:** Epithelial ovarian cancer, Tumor marker, FOXO1, PAX3

## Abstract

**Background:**

Transcription factors forkhead box protein O1 (FOXO1) and paired box 3 (PAX3) have been reported to play important roles in various cancers. However, their role in epithelial ovarian cancer (EOC) has not been elucidated yet. Therefore, we evaluated the expression and clinical significance of FOXO1 and PAX3 in EOC.

**Methods:**

Immunohistochemical analyses of FOXO1 and PAX3 in 212 EOCs, 57 borderline ovarian tumors, 153 benign epithelial ovarian tumors, and 79 nonadjacent normal epithelial tissues were performed using tissue microarray. Various clinicopathological variables, including the survival of EOC patients, were compared. In addition, the effect of FOXO1 on cell growth was assessed in EOC cell lines.

**Results:**

FOXO1 and PAX3 protein expression levels were significantly higher in EOC tissues than in nonadjacent normal epithelial tissues, benign tissues, and borderline tumors (all *p* < 0.001). In EOC tissues, FOXO1 expression was positively correlated with PAX3 expression (*Spearman’s rho* = 0.118, *p* = 0.149). Multivariate survival analysis revealed that high FOXO1 expression (hazard ratio = 2.77 [95% CI, 1.48–5.18], *p* = 0.001) could be an independent prognostic factor for overall survival. Most importantly, high expression of both FOXO1 and PAX3 showed a high hazard ratio (4.60 [95% CI, 2.00–10.55], *p* < 0.001) for overall survival. Also in vitro results demonstrated that knockdown of FOXO1 was associated with decreased cell viability, migration, and colony formation.

**Conclusions:**

This study revealed that high expression of FOXO1/PAX3 is an indicator of poor prognosis in EOC. Our results suggest the promising potential of FOXO1 and PAX3 as prognostic and therapeutic markers. The possible link between biological functions of FOXO1 and PAX3 in EOC warrants further studies.

## Background

Epithelial ovarian cancer (EOC) is the second most common gynecologic cancer and the fifth leading cause of cancer-related death in the United States of America [1]. The standard treatment for EOC is cytoreductive surgery followed by adjuvant chemotherapy. Despite significant improvements in the diagnosis and treatment of EOC, more than 70% of women are diagnosed at advanced stages, and the majority of them tend to relapse and die. Consequently, there is a great need for research to understand the molecular pathogenesis of ovarian cancer and spur the development of more specific and effective prognostic markers to improve patient outcomes.

The forkhead box (FOX) family of proteins consists of 19 sub-families of transcription factors. FOXO sub-family consists of four members, FOXO1, FOXO3, FOXO4, and FOXO6, with high protein homology [2] that are involved in diverse intracellular signaling pathways, such as phosphorylation through the phosphoinositide 3-kinas (PI3K)/protein kinase B (AKT) signaling pathway, and these FOXO members also regulate cell-cycle arrest, apoptosis, DNA damage repair, and detoxification of reactive oxygen species by regulating specific genes [3–5].

Paired box 3 (PAX3) is a member of the paired box or PAX family of transcription factors [6]. PAX3 and PAX7 comprise the group III subfamily, which share a high degree of sequence homology and similar genomic and functional organizations [7]. These transcription factors play critical roles in cell proliferation, migration, differentiation, and tissue development [8]. The role of PAX3 as an oncogene has been widely reported in various cancers, such as neuroblastoma, glioblastoma, melanoma, Ewing sarcoma, rhabdomyosarcoma, and gastric cancer [9–15].

FOXO1 is a negative regulator of the PI3K/AKT signaling pathway. Several tissue culture experiments have shown that FOXO1 is down-regulated in a wide variety of cancers, such as breast, kidney, prostate, and uterine cervix cancers [16–19]. Only a few studies have investigated FOXO1 alterations in EOC. Therefore, in this study, we evaluated the clinical significance of FOXO1 and PAX3 expressions in EOC by assessing their correlation with various clinicopathological characteristics, and performing functional studies to determine the role of FOXO1 in EOC.

## Methods

### Patients and tumor specimens

Tissue samples from 212 EOCs, 57 borderline ovarian tumors, 153 benign epithelial ovarian tumors, and 79 nonadjacent normal epitheliums were included in the study. Tissue samples were obtained from patients who underwent primary surgery at Gangnam Severance Hospital between 1996 and 2012 and the Korea Gynecologic Cancer Bank as part of Bio & Medical Technology Development program of the Ministry of the National Research Foundation (NRF) funded by the Korean government (MIST) (NRF-2017M3A9B8069610). Tumor staging was assessed according to the International Federation of Gynecology and Obstetrics (FIGO) classification. Clinical information, including age, surgical procedure, survival time, and survival status, were collected from medical records. Response Evaluation Criteria in Solid Tumors (RECIST; version 1.0) was used to assess the patients’ response to therapy by spiral computed tomography [20]. Pathological reports were reviewed to obtain tumor grade and cell types. All tumor tissues were histologically examined by one gynecologic pathologist, and all biological samples were collected after obtaining informed consent from participants, according to the guidelines of the institutional review board (IRB) of Gangnam Severance Hospital.

### Cell lines

Human ovarian cancer cell lines OVCA433 and OVCA429 were kindly gifted by Dr. Samuel C. Mok (University of Texas MD Anderson Cancer Center, Houston, USA). A polymerase chain reaction (PCR)-based method was done to test for possible contamination by mycoplasma according to the manufacturer’s instructions (*i*-MycoPCR mycoplasma detection kit; iNtRON Biotechnology Inc., Seongnam, Korea). OVCA433 and OVCA429 cells were grown in DMEM supplemented with 10% fetal bovine serum (FBS), 1% penicillin, and 1% streptomycin, and were cultured at 37 °C in a humidified atmosphere containing 5% CO_2_.

### Immunoblotting

Protein levels in OVCA433 and OVCA429 cells were measured by western blotting. OVCA433 and OVCA429 cells were homogenized in RIPA buffer (150 mM sodium chloride, 1% Triton X-100, 1% sodium deoxycholate, 0.1% SDS, 50 mM Tris-HCl, pH 7.5, and 2 mM EDTA) containing proteinase inhibitor cocktail (Roche, Nutley, NJ) to lyse the cells. Then, cell lysates were centrifuged at 13,500×*g* for 30 min, and supernatants were recovered. Lysate supernatants containing about 30 μg of protein were resolved by sodium dodecyl sulfate polyacrylamide gel electrophoresis (SDS-PAGE), and analyzed by western blotting using anti-α-tubulin (mouse antibody clone# sc-5286; Santa Cruz Biotechnology, Santa Cruz, CA) and anti-FOXO1 (mouse antibody clone# sc-374,427; Santa Cruz Biotechnology) antibodies.

### Knockdown of FOXO1 by RNA interference

Synthetic small interfering RNAs (siRNAs) specific for FOXO1 were purchased from Bioneer (Daejeon, Korea). The following sequences of FOXO1 and nonspecific (NS) siRNAs used: FOXO1 #1 sense 5′-CUGCAUAGCAUCAAGUCUU-3′ and antisense 5′-AAGACUUGTUGCUAUGCAG-3′, FOXO1 #2 sense 5′-GUCCAAGACAUAGCUGGUU-3′ and antisense 5′-AACCAGCUAUGUCUUGGACC-3′, and FOXO1 #3 sense 5′-GAGGGUUAGUGAGCAGGUU-3′ and antisense 5′-AACCUGCUCACUAACCCUC-3′. For in vitro delivery, cells in a 6-well plate were transfected with 100 pmol of siRNA using Lipofectamine™ RNAiMAX reagent (Invitrogen, Carlsbad, CA) according to the manufacturer’s instructions. The siRNA-treated cells were collected 3 days after transfection for western blot analysis.

### Cell viability assay

Control and FOXO1 siRNA-transfected cells were seeded at 1 × 10^4^ cells per well in a 96-well plate, and incubated for 1, 2, or 3 days. At each time point, cells were mixed with 10 μL of EZ-CYTOX reagent (Cat. # EZ-3000; Dogenbio, Seoul, Korea), and plates were incubated at 37 °C for 1 h. After shaking for 1 min on an orbital shaker, the absorbance was measured with a microplate reader (Bio-Rad Laboratories, Inc., Hercules, CA) at 450 nm. The experiment was performed in triplicate.

### Cell migration assay

Cell migration was assessed by Boyden chamber migration assay. OVCA433 and OVCA429 cells were seeded (1 × 10^5^ cells) in the upper chamber (8 μm polycarbonate membrane; Neuro Probe #PFB8) containing 56 μL of DMEM without FBS. DMEM supplemented with 10% FBS (27 μL) was added to the lower chamber, and the chamber was incubated for 24 h. Cells that migrated through the membrane were fixed with Diff-Quik fixative solution for 2 min, and stained with Diff-Quik staining solutions 1 and 2 for 2 min each. Then, non-migrated cells were removed with wipers, and invaded cells were counted in three random fields under Axio Imager.M2 Microscope (200× magnification; Carl Zeiss, Thornwood, NY). Each experiment was repeated three times.

### Colony formation assay

In order to examine the clonogenicity, OVCA433 and OVCA429 cells were seeded with 250 cells in a 6-wells plate and cultured in DMEM supplemented with 10% FBS for 2 weeks. Colonies formed in each well were fixed with 3.7% paraformaldehyde sucrose and stained with 0.5% crystal violet for 30 min, and then washed with distilled water. Stained cells were dissolved in 2% DMSO for 20 min on an orbital shaker, and the absorbance was measured at 595 nm. Each cell group was examined in triplicate.

### Tissue microarray and immunohistochemistry

A tissue microarray (TMA) was constructed of tissue cores (1 mm) containing sufficient proportion of tumor cells punched from formalin-fixed paraffin-embedded tumor tissue blocks. TMA blocks were cut into 5-μm-thick sections on a rotary microtome, and then deparaffinized and rehydrated in graded ethanol. Next, the sections were treated with a 3% H_2_O_2_ solution in methanol for 30 min to quench endogenous peroxidase activity. Then, heat-induced antigen retrieval was performed by incubating the sections for 20 min in target retrieval buffer at pH 6.0 (Dako, Carpinteria, CA) for FOXO1 and in a buffer at pH 9.0 for PAX3 using a steam pressure cooker (Pascal; Dako). The slides were then stained with an anti-FOXO1 antibody (rabbit antibody, clone# EP927Y, 1:400; Abcam, Cambridge, MA) and an anti-PAX3 antibody (rabbit polyclonal antibody, Cat. # Ab216683, 1:200; Abcam) for 1 h at room temperature using Autostainer Plus (Dako). Antigen-antibody reactions were visualized by using En vision^+^ Dual Link System-HRP (Dako) and DAB^+^ (3, 3′-diaminobenzidine; Dako). The stained sections were dehydrated and counterstained with hematoxylin and mounted in Faramount Aqueous Mounting Medium (Dako). Appropriate negative and positive controls were included.

### Evaluation of IHC staining

The stained TMA sections were scanned using a high-resolution optical scanner (NanoZoomer 2.0 HT; Hamamatsu Photonics K.K., Japan) at 20× objective magnification (0.5 μm resolution). The scanned sections were analyzed with Visiopharm software, version 4.5.1.324 (Hørsholm, Denmark). Brown staining intensity was scored on a scale from 0 to 3 (0 = negative, 1 = weak, 2 = moderate, and 3 = strong) using a predefined algorithm and optimized settings. The overall IHC score (histoscore) was calculated as the percentage of positive cells multiplied by staining intensity (score range: 0–300).

### Statistical analysis

Statistical analyses of FOXO1 and PAX3 expression data were performed by either Mann-Whitney or Kruskal-Wallis test, as appropriate. Kaplan-Meier method was used to assess the overall survival (OS) and disease free survival (DFS), and survival was analyzed by log-rank test using the cut-off values that showed the highest discriminative power (histoscores of 136 for FOXO1 and 156 for PAX3). Cox proportional hazards model was used to estimate the hazard ratios and confidence intervals (CIs) in both univariate and multivariate models. Statistical analysis was performed using SPSS version 23.0 (SPSS, Inc., Chicago, IL). *P*-value less than 0.05 was considered statistically significant.

## Results

### FOXO1 and PAX3 expression levels were elevated in EOC tissues

**Fig. 1:**
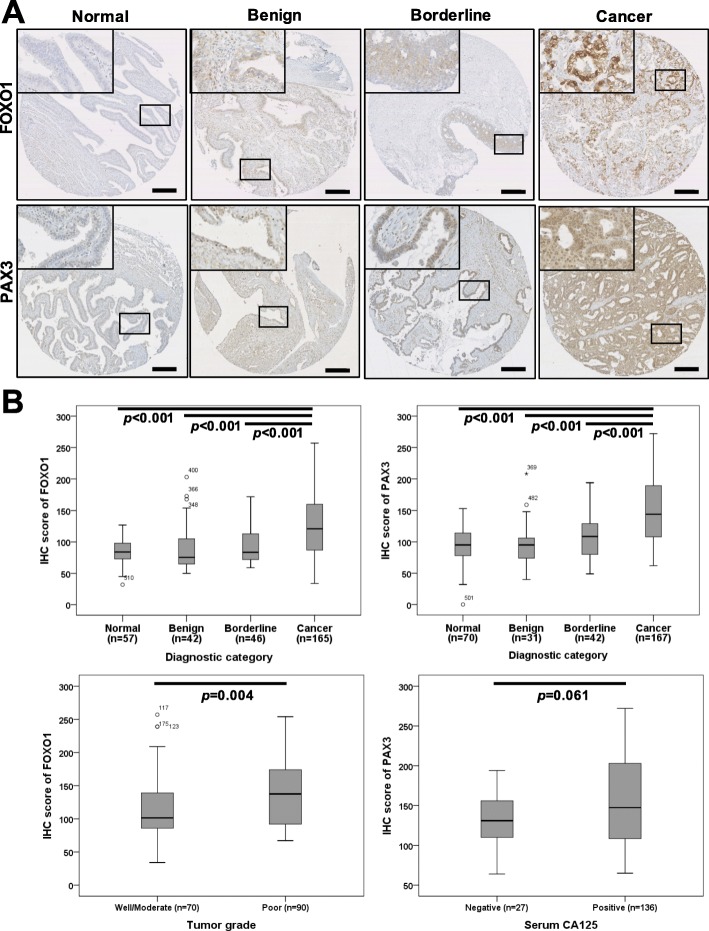
Immunohistochemical (IHC) staining of FOXO1 and PAX3 in epithelial ovarian cancer samples, **a** Representative images of IHC staining for FOXO1 and PAX3 in normal, benign, and borderline tumors and epithelial ovarian cancer (Cancer) tissue samples (scale bar: 50 μm). **b** Boxplots of IHC staining data (histoscores) according to various clinicopathological characteristics. Histoscores were calculated based on staining intensity and the area of positive staining

**Fig. 2:**
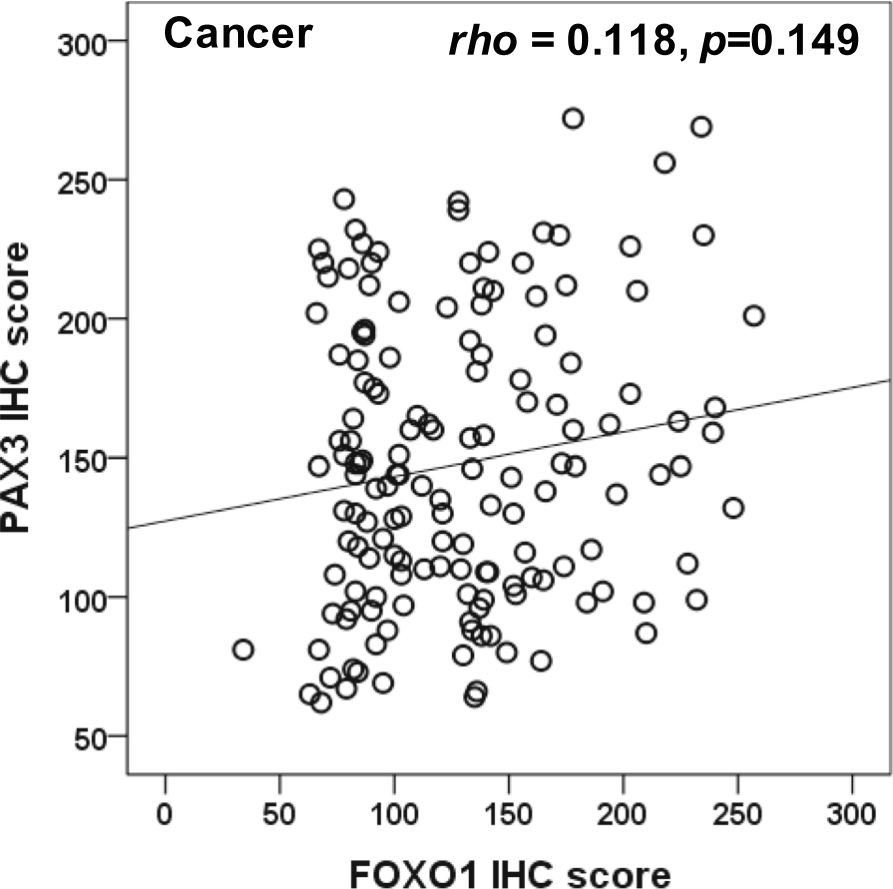
Correlation between the expression levels of FOXO1 and PAX3. FOXO1 expression showed a tendency to be positively correlated with PAX3 expression in EOC. (*Spearman’s rho = 0.118*, *p* = 0.149)

To evaluate the protein expressions of FOXO1 and PAX3 in EOC, we analyzed FOXO1 and PAX3 protein levels in 212 EOC tissues, 57 borderline tumors, 153 benign tumors, and 79 nonadjacent normal epithelial tissues by IHC. Some samples were lost due to problems with the sectioning and staining of samples. Finally, FOXO1 data from 165 EOC tissues, 46 borderline tumors, 42 benign tumors, and 57 nonadjacent normal epithelial tissues, as well as PAX3 data from 167 EOC tissues, 42 borderline tumors, 31 benign tumors, and 70 nonadjacent normal epithelial tissues could be interpreted. Most FOXO1 immunoreactivity was observed in the cytoplasm, while most PAX3 immunoreactivity was in the nucleus (Fig. 1a). IHC scores for FOXO1 and PAX3 are summarized in Table 1, and data revealed that FOXO1 and PAX3 expression levels were significantly higher in EOC tissues than in borderline tumors (both *p* < 0.001), benign tumors (both *p* < 0.001), and nonadjacent normal epithelial tissues (both *p* < 0.001; Fig. 1b). In addition, FOXO1 immunoreactivity was positively correlated with poor tumor grade (*p* = 0.004; Fig. 1b). Although not significant, there was a trend towards a positive correlation between PAX3 immunoreactivity and CA125, as shown in Fig. 1b and Table 1 (*p* = 0.061). Next, we examined the association between FOXO1 and PAX3 expression. Spearman’s rank correlation analysis revealed that FOXO1 expression tended to be positively correlated with PAX3 expression in EOC (*Spearman’s rho* = 0.118, *p* = 0.149; Fig. 2).

**Table 1:** Expressions of FOXO1 and PAX3 in relation to clinicopathological characteristics in IHC analysis

	No.	FOXO1		No.	PAX3	
		Mean score (95% CI)	*p-value*		Mean score (95% CI)	*p-value*
All study subjects	310	110.9 [105.7–116.0]		310	125.2 [119.7–130.8]	
Diagnostic category						
Normal	57	85.3 [79.9–90.8]	*< 0.001*	70	93.6 [87.2–100.0]	*< 0.001*
Benign	42	90.3 [78.7–101.8]		31	95.2 [82.3–108.1]	
Borderline	46	93.6 [85.4–101.9]		42	107.0 [96.1–117.8]	
Cancer	165	129.8 [122.1–137.5]		167	148.7 [140.9–156.5]	
FIGO stage			*0.854*			*0.230*
I-II	43	131.1 [115.2–147.0]		43	143.3 [130.8–155.8]	
III-IV	106	132.8 [123.3–142.3]		107	154.1 [143.9–164.2]	
Cell type			*0.996*			*0.319*
Serous	120	129.8 [121.0–138.6]		122	151.1 [141.6–160.6]	
Others	45	129.7 [113.6–145.8]		45	142.2 [128.5–155.9]	
Tumor grade			*0.004*			*0.365*
Well/Moderate	70	118.1 [106.9–129.2]		70	153.7 [141.3–166.0]	
Poor	90	140.8 [130.1–151.4]		89	146.2 [135.3–157.0]	
CA125			*0.470*			*0.061*
Negative	25	137.3 [116.2–158.4]		27	133.0 [119.1–147.0]	
Positive	137	129.5 [121.1–137.8]		136	153.0 [144.1–161.9]	
Chemosensitivity			*0.112*			*0.254*
Sensitive	144	129.6 [121.4–137.9]		147	149.6 [141.4–157.9]	
Resistant	10	156.0 [116.0–195.9]		10	130.4 [86.3–174.4]	

*FIGO* International Federation of Gynecology and Obstetrics, *CI* Confidence interval

### FOXO1 overexpression is associated with poor prognosis

**Fig. 3:**
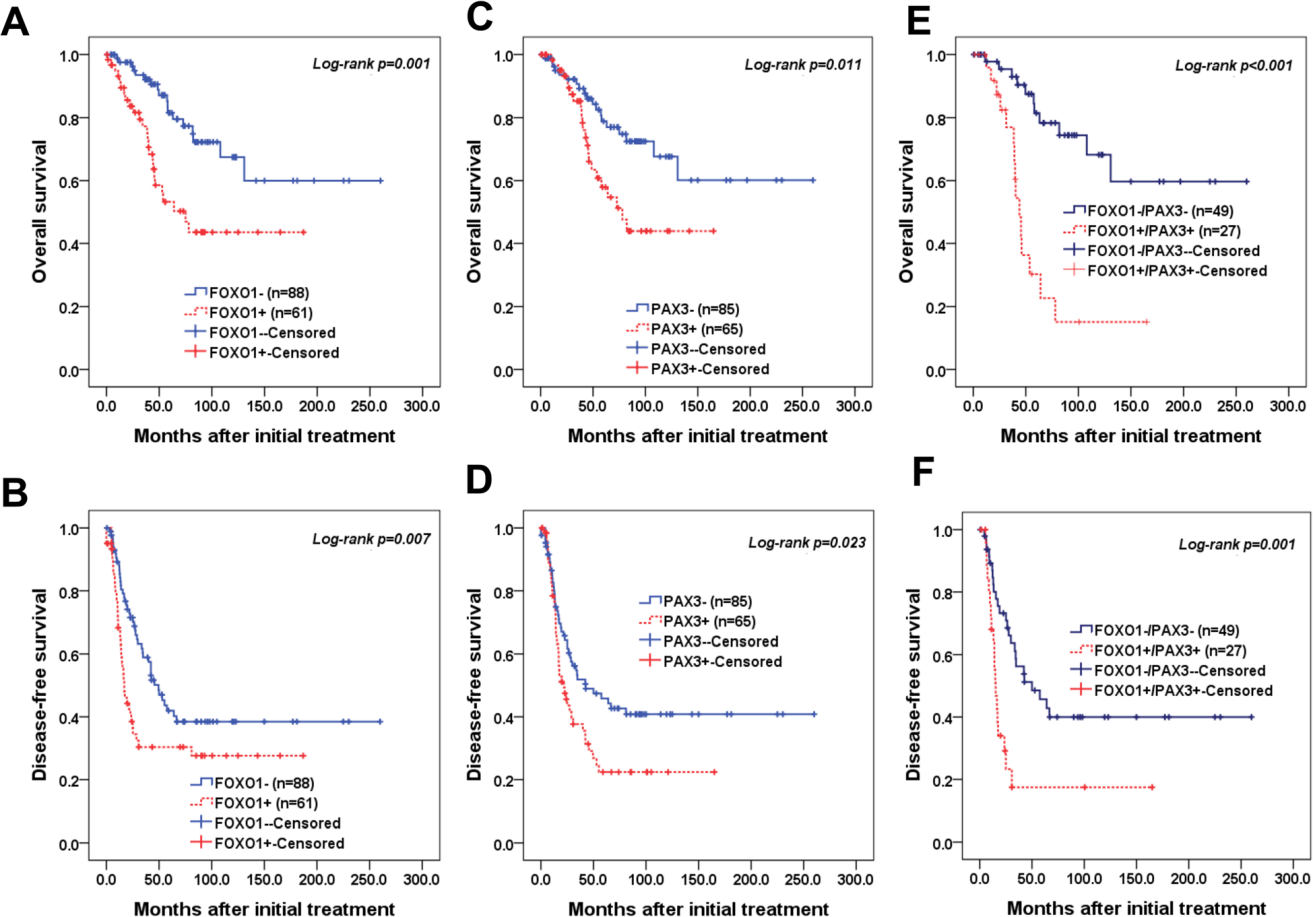
Kaplan-Meier survival curves of patients with epithelial ovarian cancer. Epithelial ovarian cancer (EOC) patients with FOXO1+ (histoscore > 137) and PAX3+ (histoscore > 156) tumors showed significantly worse (**a, c**) overall survival (*p = 0.001* and *p = 0.011*, respectively) and (**b, d**) disease-free survival (*p = 0.007* and *p = 0.023*, respectively) compared to patients with FOXO1- and PAX3- tumors. Patients with high FOXO1/PAX3 expressions had worse (**e**) overall survival (*p* < 0.001) and (**f**) disease-free survival (*p* = 0.001).

We then examined the relationship between FOXO1 expression and outcomes in EOC patients. OS and DFS were analyzed by Kaplan-Meier plots to determine the relationship between FOXO1 and PAX3 expression and survival (Fig. 3). Survival analysis included 165 EOC patients for FOXO1 and 167 EOC patients for PAX3 who underwent optimal debulking surgery. Kaplan-Meier plots demonstrated that patients with high FOXO1 expression (cut-off value: 137) or high PAX3 expression (cut-off value: 156) displayed significantly poorer OS (log-rank *p* = 0.001 and *p* = 0.011, respectively; Fig. 3a, c). DFS analysis showed significant survival disadvantages in patients with high FOXO1 or PAX3 expression (Log-rank *p* = 0.007 and *p* = 0.023, respectively; Fig. 3b, d). Furthermore, significant differences in both OS (*p* < 0.001; Fig. 3e) and DFS (*p* = 0001; Fig. 3f) were observed for patients with high expressions of both FOXO1 and PAX3 compared to patients with low expression levels. Cox multivariate proportional hazards analysis showed that high FOXO1 expression (hazard ratio = 2.77 [95% CI, 1.48–5.18], *p* = 0.001) was an independent prognostic factor for poor OS (Table 2). Notably, high expressions of both FOXO1 and PAX3 were strong independent prognostic factors for poor OS (hazard ratio = 4.60 [95% CI, 2.00–10.55], *p* < 0.001; Table 2), and high FOXO1 expression was an independent poor prognostic factor for DFS (hazard ratio = 1.71 [95% CI, 1.05–2.78], *p* = 0.029; Table 2).

**Table 2:** Univariate and multivariate analyses of the associations between prognostic variables and overall and disease-free survival rates in epithelial ovarian cancer

	Overall survival hazard ratio [95% CI*], *p*-value		Disease-free survival hazard ratio [95% CI*], *p*-value	
	Univariate	Multivariate	Univariate	Multivariate
FIGO stage (III-IV)	3.86 [1.52–9.82], 0.004	3.04 [1.15–8.04], 0.025	5.59 [2.79–11.20], < 0.001	4.98 [2.30–10.79], < 0.001
Cell type (serous)	3.43 [1.35–8.73], 0.009	2.24 [0.84–5.93], 0.105	3.19 [1.77–5.77], < 0.001	2.14 [1.00–4.58], 0.049
Tumor grade (poor)	1.79 [0.96–3.31], 0.064	NA	2.08 [1.34–3.23], 0.001	1.28 [0.77–2.11], 0.333
CA125+ (> 35 U/mL)	1.74 [0.62–4.89], 0.290	NA	1.92 [0.96–3.83], 0.064	NA
Age (> 50)	1.79 [0.96–3.35], 0.067	NA	1.34 [0.87–2.05], 0.173	NA
FOXO1 + ^a^	2.74 [1.49–5.04], 0.001	2.77 [1.48–5.18], 0.001	1.79 [1.16–2.76], 0.008	1.71 [1.05–2.78], 0.029
PAX3 + ^b^	2.17 [1.17–4.01], 0.013	1.56 [0.82–2.93], 0.168	1.62 [1.06–2.47], 0.025	1.00 [0.64–1.57], 0.980
FOXO1+/PAX3+	5.53 [2.47–12.40], < 0.001	4.60 [2.00–10.55], < 0.001	2.75 [1.47–5.15], 0.001	1.84 [0.97–3.50], 0.061

*CI*^†^ Confidence interval; *NA* Not applicable

^a^Cut-off value of FOXO1^+^ was over 137 of IHC score; ^b^cut-off of PAX3^+^ was over 156 of IHC score; *CI Confidence interval, *FIGO* International Federation of Gynecology and Obstetrics, *LN* Lymph node; *NA* Not applicable

### Knockdown of FOXO1 in ovarian cancer cells decreased cell viability, migration, and colony formation

**Fig. 4:**
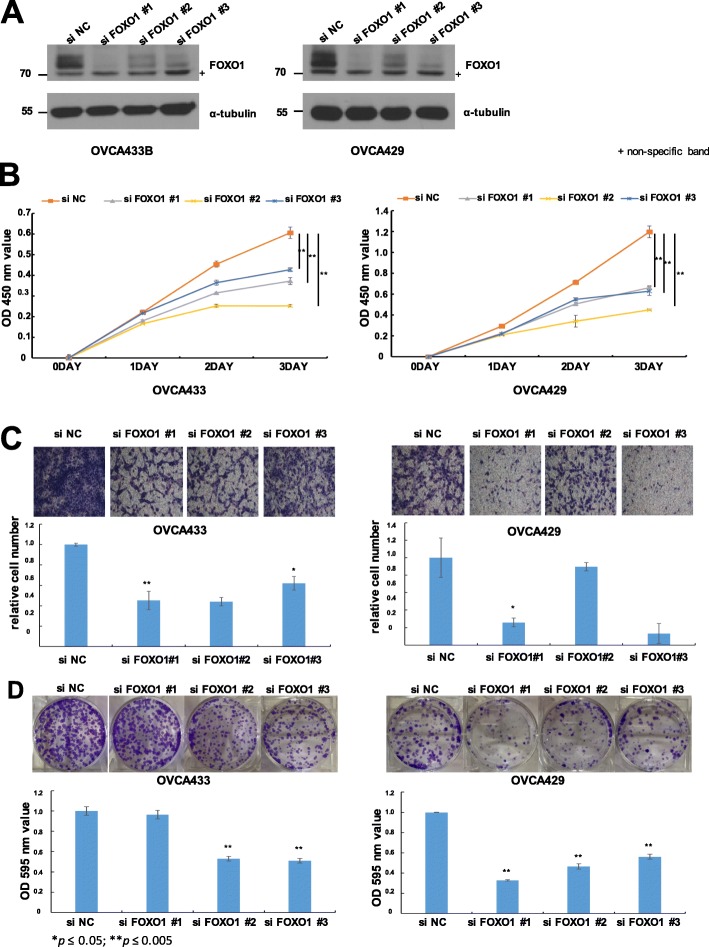
Effect of FOXO1 on the proliferation and migration of OVCA433 and OVCA429 cells. **a** Western blot analysis of FOXO1 expression following siRNA (siFOXO1)-transfection induced knockdown in OVCA433 and OVCA429 cells. FOXO1 protein expression was decreased compared to the levels of negative control (siNC) cells. **b** Curves of the viability of siNC- and siFOXO1-transfected OVCA433 and OVCA429 cells collected at different time points. The viability of FOXO1 knockdown cells decreased. **c** Cell migration assay of siNC- and siFOXO1-transfected OVCA433 and OVCA429 cells. Upper panel: representative images of migrated cells. Lower panel: quantitative results of cell migration experiments. The assay showed that FOXO1 knockdown (siFOXO1-transfected cells) resulted in decreased migration and invasion compared to negative control (siNC) cells. **d** Colonogenic assay performed on OVCA433 and OVCA429 cells. Upper panel: representative figures of colonogenic assay. Lower panel: quantitative results of colonogenic assay. Colony formation decreased in FOXO1 knockdown (siFOXO) compared to negative control (siNC). The number of asterisks (*) indicates the level of significance: *p ≤ 0.05, **p ≤ 0.005. Data and error bars represent the mean ± SD of triplicate experiments.

Maintaining proliferation, escaping growth suppression, and migration are three qualities that a normal cell must acquire to become a cancer cell. Therefore, to elucidate the biological functions of FOXO1 in EOC cells, we modulated intracellular FOXO1 expression in OVCA433 and OVCA429 cells and assessed cell proliferation and migration. OVCA433 and OVCA429 cells were transfected with either a FOXO1 knockdown (FOXO1 siRNA) vector or a control vector (no insert), and FOXO1 expression levels in transfected cells were measured by western blotting (Fig. 4a). To evaluate the proliferation ability, we analyzed the short-term and long-term effects by cell viability assay and colony forming assay. Cell growth was significantly lower in both FOXO1 siRNA-transfected cell lines than in negative control (siNC)-transfected cells (Fig. 4b). Also, colony formation was significantly decreased in both FOXO1 siRNA-transfected cell lines than in siNC-transfected cells (Fig. 4d). To investigate whether FOXO1 plays a role in the migration of EOC cells, we conducted Boyden chamber assay. The siRNA-mediated FOXO1 knockdown significantly altered cell migration compared to that of siNC- transfected cells (Fig. 4c). These data showed that FOXO1 plays a key role in the proliferation and migration of EOC cells.

## Discussion

Despite significant improvements in the treatment of EOC, the mortality rate still remains high. Understanding the molecular mechanisms related to the progression and metastasis of EOC is necessary for the development of useful prognostic biomarkers. Transcription factor FOXO1 plays an important role in glucose metabolism, cell cycle progression, apoptosis, and differentiation [3]. Although the functions of FOXO1 have not been fully elucidated, it has been shown to act as a tumor suppressor in various human cancers, including melanoma as well as prostate, bladder, and breast cancers [21–23]. However, the role of FOXO1 in the carcinogenesis of EOC is controversial. To gain insight into the role of FOXO1 in EOC and its relationship with PAX3, we performed IHC to detect FOXO1 and PAX3 in EOC tissues.

Our IHC analysis showed that FOXO1 is significantly upregulated in EOC tissues compared to borderline tumors, benign tumors, and nonadjacent normal epithelium (all *p* < 0.001, Table 1). In contrast to our results, FOXO1 is downregulated in tumors from other organs, such as the uterine cervix, kidney, breast, prostate, and endometrium [16–19, 24]. Wang et al. reported that FOXO1 is downregulated in EOC [24]. This discrepancy in the expression status of FOXO1 might be attributed to differences in methodology-related factors, such as IHC techniques, antibodies, and samples, as well as interobserver variations.

Next, the relationship between FOXO1 expression, as determined by IHC, and clinicopathological factors in EOC patients, including survival outcomes, was investigated. Cox multivariate analysis showed that FOXO1 is an independent poor prognostic factor for OS and DFS in EOC, which means that upregulation of FOXO1 in the cytoplasm follows malignant transformation in the carcinogenesis of in EOC patients, and is associated with poor prognosis. Similar to our study, a number of other studies have shown that high FOXO expression and/or activity is correlated with poor prognosis, whereas many other studies have shown that high expression and/or nuclear localization of FOXO is correlated with favorable prognosis [25–38]. As previously mentioned, Wang et al. reported that high FOXO1 expression in EOC was positively correlated with a good prognosis, which is the opposite of our results [39]. These contradicting results could be due to differences between the two studies. First, Wang et al. analyzed survival data from the Cancer Genome Atlas (TCGA) database. TCGA data are quantitative mRNA expression levels, while our data are protein levels as determined by IHC analysis. Since many cellular processes after transcription are ultimately regulated by protein levels, there is a strong possibility of discrepancies between the two studies. Moreover, Wang et al. included only ovarian serous cystadenocarcinoma patients, while we included different histopathological subtypes, such as serous, mucinous, and endometrioid.

Since our analysis of clinical specimens suggested a critical role for FOXO1 in tumorigenesis, we examined the role of FOXO1 in tumor cell growth (proliferation) and metastasis (migration) in functional studies. In cell viability assay, siRNA-mediated FOXO1 knockdown in OVCA433 and OVCA429 cells led to decreased cell proliferation. In addition, migration assay showed that FOXO1 knockdown had negative significant effects on the migration of EOC cells. PI3/AKT signaling pathway is a key mechanism for the role of FOXO family members in various cancers. It has been shown that PI3K/AKT pathway negatively regulates the transcriptional activity of FOXO1, FOXO3, and FOXO4; however, when FOXO3 was coactivated with β-catenin, FOXO3 showed pro-tumoral activity [40]. In addition, it was reported that matrix metalloproteinase-1 (MMP-1) is induced by FOXO1 and enhances the invasive potential of human breast cancer cells [41]. Therefore, further molecular studies are needed to determine the detailed mechanism by which FOXO1 confers growth and invasion advantages to EOC cells.

To determine the mechanism underlying the role of FOXO1 in tumorigenesis, we focused on the correlation between FOXO1 and PAX3 in EOC, since PAX3-FOXO1 is a well-known fusion protein that is associated with alveolar rhabdomyosarcoma and modulates multiple signaling pathways involved in cell proliferation, migration, and death. Before evaluating the correlation between FOXO1 and PAX3, PAX3 expression in EOC was analyzed by IHC to determine the role of PAX3 in EOC. The results showed that overexpression of PAX3 in EOC tissues was significantly associated with poor prognosis, as reported in previous studies on melanoma, glioma, gastric carcinoma, and breast cancer [12, 42–44]. Multivariate analysis showed that upregulation of both FOXO1 and PAX3 is a strong independent prognostic factor for OS. We also observed a non-significant trend toward a positive correlation between FOXO1 and PAX3. To our knowledge, there are only a few existing studies on the correlation between FOXO1 and PAX3. However, based on previous reports, there are some possible mechanisms to consider. First, FOXO1 and PAX3 might bind directly, as Kubic et al. showed that overexpression of FOXD3, a member of the FOX protein family, upregulated PAX3 expression in melanoma cells by the direct binding of FOXD3 to PAX3 promoter [45]. Second, there might be other target genes that are activated or repressed by FOXO1 and PAX3. For example, activation of P13K/AKT was enhanced upon activation of FOXO1 in renal tumors and mammalian cells [46, 47], and Liu et al. reported that PAX3 was regulated by PI3K/AKT signaling pathways in thyroid cancer [48]. FOXO1 is a well-known transcription factor that is activated in the nucleus, and its neo-cytoplasmic shuttling requires the balancing of a multitude of post-translational modifications. Furthermore, the localization of FOXO is roughly inversely correlated to PI3K activity [49]. Activation of FOXO1 and PAX3 is a dynamic process. Therefore, determining the target genes of FOXO1 and PAX3 and the localization of other FOXOs would be useful for determining the functions of FOXO1 and its relationship with PAX3. Investigating the mechanisms related to the cellular activity of FOXO1 and PAX3 will likely contribute to the development of cancer therapies.

## Conclusions

In summary, we investigated the role of FOXO1, both alone and in combination with PAX3 in EOC. Our results showed that FOXO1 was an independent prognostic factor for OS and DFS in EOC. Furthermore, high expressions of both FOXO1 and PAX3 were independent predictors of poor prognosis. PAX3 is very informative by itself, and based on this study the combination with FOXO1 and PAX3 may improve the prognostic classification of EOC.

## Data Availability

Data supporting the conclusions of this study are included in the article. Additionally, data are available to interested researchers upon reasonable request.

## Abbreviations

AKT: Protein Kinase B
CI: Confidence interval
DFS: Disease-free survival
EOC: Epithelial ovarian cancer
FOXO1: Forkhead box protein O1
IHC: Immunohistochemistry
OS: Overall survival
PAX3: Paired box3
PI3K: Phosphoinositide 3-kinas
RECIST: Response evaluation criteria in solid tumors
siRNAs: Synthetic small interfering RNAs
TCGA: The Cancer Genome Atlas
TMA: Tissue microarray
FIGO: International Federation of Gynecology and Obstetrics
H&E: Hematoxylin and eosin

## References

[1] Jemal A, Siegel R, Xu J, Ward E (2010). Cancer statistics, 2010. CA Cancer J Clin.

[2] Fu Z, Tindall DJ (2008). FOXOs, cancer and regulation of apoptosis. Oncogene.

[3] Xing YQ, Li A, Yang Y, Li XX, Zhang LN, Guo HC (2018). The regulation of FOXO1 and its role in disease progression. Life Sci.

[4] Wang Y, Zhou Y, Graves DT (2014). FOXO transcription factors: their clinical significance and regulation. Biomed Res Int.

[5] Lu H, Huang H (2011). FOXO1: a potential target for human diseases. Curr Drug Targets.

[6] Boudjadi S, Chatterjee B, Sun W, Vemu P, Barr FG (2018). The expression and function of PAX3 in development and disease. Gene.

[7] Stuart ET, Kioussi C, Gruss P (1994). Mammalian Pax genes. Annu Rev Genet.

[8] Schafer BW (1998). Emerging roles for PAX transcription factors in cancer biology. Gen Physiol Biophys.

[9] Li CG, Eccles MR (2012). PAX genes in Cancer; friends or foes?. Front Genet.

[10] Xia L, Huang Q, Nie D, Shi J, Gong M, Wu B, Gong P, Zhao L, Zuo H, Ju S (2013). PAX3 is overexpressed in human glioblastomas and critically regulates the tumorigenicity of glioma cells. Brain Res.

[11] Fang WH, Wang Q, Li HM, Ahmed M, Kumar P, Kumar S (2014). PAX3 in neuroblastoma: oncogenic potential, chemosensitivity and signalling pathways. J Cell Mol Med.

[12] Plummer RS, Shea CR, Nelson M, Powell SK, Freeman DM, Dan CP, Lang D (2008). PAX3 expression in primary melanomas and nevi. Mod Pathol.

[13] Frascella E, Toffolatti L, Rosolen A (1998). Normal and rearranged PAX3 expression in human rhabdomyosarcoma. Cancer Genet Cytogenet.

[14] Schulte TW, Toretsky JA, Ress E, Helman L, Neckers LM (1997). Expression of PAX3 in Ewing's sarcoma family of tumors. Biochem Mol Med.

[15] Zhang L, Xia L, Zhao L, Chen Z, Shang X, Xin J, Liu M, Guo X, Wu K, Pan Y (2015). Activation of PAX3-MET pathways due to miR-206 loss promotes gastric cancer metastasis. Carcinogenesis.

[16] Guttilla IK, White BA (2009). Coordinate regulation of FOXO1 by miR-27a, miR-96, and miR-182 in breast cancer cells. J Biol Chem.

[17] Zhang B, Gui LS, Zhao XL, Zhu LL, Li QW (2015). FOXO1 is a tumor suppressor in cervical cancer. Genet Mol Res.

[18] Kojima T, Shimazui T, Horie R, Hinotsu S, Oikawa T, Kawai K, Suzuki H, Meno K, Akaza H, Uchida K (2010). FOXO1 and TCF7L2 genes involved in metastasis and poor prognosis in clear cell renal cell carcinoma. Genes Chromosom Cancer.

[19] Fendler A, Jung M, Stephan C, Erbersdobler A, Jung K, Yousef GM (2013). The antiapoptotic function of miR-96 in prostate cancer by inhibition of FOXO1. PLoS One.

[20] Therasse P, Arbuck SG, Eisenhauer EA, Wanders J, Kaplan RS, Rubinstein L, Verweij J, Van Glabbeke M, van Oosterom AT, Christian MC (2000). New guidelines to evaluate the response to treatment in solid tumors. European Organization for Research and Treatment of Cancer, National Cancer Institute of the United States, National Cancer Institute of Canada. J Natl Cancer Inst.

[21] Coomans de Brachene A, Demoulin JB (2016). FOXO transcription factors in cancer development and therapy. Cell Mol Life Sci.

[22] Li R, Erdamar S, Dai H, Wheeler TM, Frolov A, Scardino PT, Thompson TC, Ayala GE (2007). Forkhead protein FKHR and its phosphorylated form p-FKHR in human prostate cancer. Hum Pathol.

[23] Zhang Y, Jia L, Zhang Y, Ji W, Li H (2017). Higher expression of FOXOs correlates to better prognosis of bladder cancer. Oncotarget.

[24] Goto T, Takano M, Albergaria A, Briese J, Pomeranz KM, Cloke B, Fusi L, Feroze-Zaidi F, Maywald N, Sajin M (2008). Mechanism and functional consequences of loss of FOXO1 expression in endometrioid endometrial cancer cells. Oncogene.

[25] Dansen TB, Burgering BM (2008). Unravelling the tumor-suppressive functions of FOXO proteins. Trends Cell Biol.

[26] Chung SY, Huang WC, Su CW, Lee KW, Chi HC, Lin CT, Chen ST, Huang KM, Tsai MS, Yu HP, et al. FoxO6 and PGC-1alpha form a regulatory loop in myogenic cells. Biosci Rep. 2013;33(3):485-97. 10.1042/BSR20130031 PMC3681252 23639108

[27] Renault VM, Thekkat PU, Hoang KL, White JL, Brady CA, Kenzelmann Broz D, Venturelli OS, Johnson TM, Oskoui PR, Xuan Z (2011). The pro-longevity gene FoxO3 is a direct target of the p53 tumor suppressor. Oncogene.

[28] Gan B, Lim C, Chu G, Hua S, Ding Z, Collins M, Hu J, Jiang S, Fletcher-Sananikone E, Zhuang L (2010). FoxOs enforce a progression checkpoint to constrain mTORC1-activated renal tumorigenesis. Cancer Cell.

[29] Bouchard C, Lee S, Paulus-Hock V, Loddenkemper C, Eilers M, Schmitt CA (2007). FoxO transcription factors suppress Myc-driven lymphomagenesis via direct activation of Arf. Genes Dev.

[30] Habashy HO, Rakha EA, Aleskandarany M, Ahmed MA, Green AR, Ellis IO, Powe DG (2011). FOXO3a nuclear localisation is associated with good prognosis in luminal-like breast cancer. Breast Cancer Res Treat.

[31] Hillion J, Le Coniat M, Jonveaux P, Berger R, Bernard OA (1997). AF6q21, a novel partner of the MLL gene in t(6;11)(q21;q23), defines a forkhead transcriptional factor subfamily. Blood.

[32] Kim SY, Yoon J, Ko YS, Chang MS, Park JW, Lee HE, Kim MA, Kim JH, Kim WH, Lee BL (2011). Constitutive phosphorylation of the FOXO1 transcription factor in gastric cancer cells correlates with microvessel area and the expressions of angiogenesis-related molecules. BMC Cancer.

[33] Kim JH, Kim MK, Lee HE, Cho SJ, Cho YJ, Lee BL, Lee HS, Nam SY, Lee JS, Kim WH (2007). Constitutive phosphorylation of the FOXO1A transcription factor as a prognostic variable in gastric cancer. Mod Pathol.

[34] Santamaria CM, Chillon MC, Garcia-Sanz R, Perez C, Caballero MD, Ramos F, de Coca AG, Alonso JM, Giraldo P, Bernal T (2009). High FOXO3a expression is associated with a poorer prognosis in AML with normal cytogenetics. Leuk Res.

[35] Chen J, Gomes AR, Monteiro LJ, Wong SY, Wu LH, Ng TT, Karadedou CT, Millour J, Ip YC, Cheung YN (2010). Constitutively nuclear FOXO3a localization predicts poor survival and promotes Akt phosphorylation in breast cancer. PLoS One.

[36] Hagenbuchner J, Rupp M, Salvador C, Meister B, Kiechl-Kohlendorfer U, Muller T, Geiger K, Sergi C, Obexer P, Ausserlechner MJ (2016). Nuclear FOXO3 predicts adverse clinical outcome and promotes tumor angiogenesis in neuroblastoma. Oncotarget.

[37] Corno C, Stucchi S, De Cesare M, Carenini N, Stamatakos S, Ciusani E, Minoli L, Scanziani E, Argueta C, Landesman Y (2018). FoxO-1 contributes to the efficacy of the combination of the XPO1 inhibitor selinexor and cisplatin in ovarian carcinoma preclinical models. Biochem Pharmacol.

[38] Beretta Giovanni Luca, Corno Cristina, Zaffaroni Nadia, Perego Paola (2019). Role of FoxO Proteins in Cellular Response to Antitumor Agents. Cancers.

[39] Wang Z, Ji G, Wu Q, Feng S, Zhao Y, Cao Z, Tao C (2018). Integrated microarray meta-analysis identifies miRNA-27a as an oncogene in ovarian cancer by inhibiting FOXO1. Life Sci.

[40] Tenbaum Stephan P, Ordóñez-Morán Paloma, Puig Isabel, Chicote Irene, Arqués Oriol, Landolfi Stefania, Fernández Yolanda, Herance José Raúl, Gispert Juan D, Mendizabal Leire, Aguilar Susana, Cajal Santiago Ramón y, Schwartz Simó, Vivancos Ana, Espín Eloy, Rojas Santiago, Baselga José, Tabernero Josep, Muñoz Alberto, Palmer Héctor G (2012). β-catenin confers resistance to PI3K and AKT inhibitors and subverts FOXO3a to promote metastasis in colon cancer. Nature Medicine.

[41] Feng X, Wu Z, Wu Y, Hankey W, Prior TW, Li L, Ganju RK, Shen R, Zou X (2011). Cdc25A regulates matrix metalloprotease 1 through Foxo1 and mediates metastasis of breast cancer cells. Mol Cell Biol.

[42] Muratovska A, Zhou C, He S, Goodyer P, Eccles MR (2003). Paired-box genes are frequently expressed in cancer and often required for cancer cell survival. Oncogene.

[43] Staron MM, Gray SM, Marshall HD, Parish IA, Chen JH, Perry CJ, Cui G, Li MO, Kaech SM (2014). The transcription factor FoxO1 sustains expression of the inhibitory receptor PD-1 and survival of antiviral CD8(+) T cells during chronic infection. Immunity.

[44] Xu Y, Shao QS, Yao HB, Jin Y, Ma YY, Jia LH (2014). Overexpression of FOXC1 correlates with poor prognosis in gastric cancer patients. Histopathology.

[45] Kubic JD, Little EC, Kaiser RS, Young KP, Lang D (2016). FOXD3 promotes PAX3 expression in melanoma cells. J Cell Biochem.

[46] Lin A, Piao HL, Zhuang L, Sarbassov dos D, Ma L, Gan B (2014). FoxO transcription factors promote AKT Ser473 phosphorylation and renal tumor growth in response to pharmacologic inhibition of the PI3K-AKT pathway. Cancer Res.

[47] Chen CC, Jeon SM, Bhaskar PT, Nogueira V, Sundararajan D, Tonic I, Park Y, Hay N (2010). FoxOs inhibit mTORC1 and activate Akt by inducing the expression of Sestrin3 and Rictor. Dev Cell.

[48] Liu W, Sui F, Liu J, Wang M, Tian S, Ji M, Shi B, Hou P (2016). PAX3 is a novel tumor suppressor by regulating the activities of major signaling pathways and transcription factor FOXO3a in thyroid cancer. Oncotarget.

[49] Kim HJ, Lee SY, Kim CY, Kim YH, Ju W, Kim SC (2017). Subcellular localization of FOXO3a as a potential biomarker of response to combined treatment with inhibitors of PI3K and autophagy in PIK3CA-mutant cancer cells. Oncotarget.

